# Diagnostic Model Incorporating Clinicopathological Characteristics of Delphian Lymph Node Metastasis Risk Profiles in Papillary Thyroid Cancer

**DOI:** 10.3389/fendo.2021.591015

**Published:** 2021-03-25

**Authors:** Xingchen Li, Yuansheng Duan, Dandan Liu, Hongwei Liu, Mengqian Zhou, Kai Yue, Yanjie Shuai, Yu Wang, Chenyan Ji, Chao Jing, Yansheng Wu, Xudong Wang

**Affiliations:** ^1^Department of Maxillofacial & E.N.T. Oncology, Tianjin Medical University Cancer Institute & Hospital, Key Laboratory of Cancer Prevention and Therapy, Tianjin Cancer Institute, National Clinical Research Center of Cancer, Tianjin, China; ^2^Department of Epidemiology and Biostatistics, School of Public Health, Tianjin Medical University, Tianjin, China

**Keywords:** risk factor, nomogram, diagnostic model, papillary thyroid cancer, Delphian lymph node

## Abstract

The Delphian lymph node (DLN), also known as the prelaryngeal node, is one component of the central lymph node. The DLN has been well studied in laryngeal cancer, although its significance in papillary thyroid cancer (PTC) remains unclear. We retrospectively analyzed 936 patients with PTC who underwent thyroidectomy by a single surgeon in Tianjin Cancer Hospital from 2017 to 2019. Moreover, 250 PTC patients who underwent thyroidectomy by another surgeon in Tianjin Cancer Hospital from January 2019 to April 2019 were used as a validation cohort. Among the 936 patients with PTC, 581 patients (62.1%) had DLNs, of which 177 samples with metastasis (177/581, 30.5%) were verified. DLN metastasis was significantly correlated with sex, age, tumor size, bilateral cancer, multifocality, extrathyroidal extension, lymphovascular invasion and central and lateral neck lymph node metastasis. Multivariate analysis revealed that independent risk factors for DLN metastasis included age, gender, tumor size, extrathyroid extension, lymphovascular invasion and central lymph node metastasis, which determined the nomogram. In particular, tumor size was proven to be one of the most predominant single predictors. The diagnostic model had an area under the curve (AUC) of 0.829 (95% confidence interval, 0.804–0.854). The internal and external validations of the nomogram were 0.819 and 0.745, respectively. Our results demonstrate that DLN metastasis appears to be a critical parameter for predicting metastatic disease of the central compartments. Furthermore, this study provides a precise criterion for assessing DLN metastasis and has great clinical significance for treating PTC.

## Introduction

Thyroid neoplasms are the fifth most prevalent malignancy in women and account for 3% of all human malignancies (1). Papillary thyroid carcinoma (PTC) is the most common histopathological subtype, accounting for approximately 90% of all thyroid carcinomas (2). Although the 20-year overall survival rate of PTC reaches up to 90% and its disease-specific survival rate is approximately 97% (3), metastasis to regional cervical lymph nodes occurs early and frequently, and the rate of lymph node metastasis is as high as 90% in PTC (4). The central neck nodes are the most common sites of nodal metastasis in PTC patients (5, 6). Emerging evidence has suggested that cervical lymph node metastasis acts as an unfavorable factor that contributes to locoregional recurrence (7–9).

The DLN, also known as the prelaryngeal or precricoid lymph node, usually consists of a single node or a group of lymph nodes and receives lymphatic drainage from the larynx and thyroid (10, 11). The Delphian, pretreacheal, and paratracheal lymph node groups compose the central neck lymph nodes. Increasing evidence indicates that DLN metastasis is an effective predictor of regional lymph node disease and recurrence in many malignant head and neck cancers, including PTC (12–16). Currently, the role of DLN metastasis in PTC has gained substantial attention (17–19). Reports have indicated that DLN metastasis is an aggressive disease and predicts a high risk of recurrence in PTC (14, 20). At the moment, it is controversial whether clinically lymph node-negative (cN0) PTC patients should undergo prophylactic central lymph node dissection. The DLN, one component of central neck node clusters, may be an available preoperative indicator to help surgeons to make individualized treatment plans. Hence, a high-efficiency DLN metastasis evaluation system will be of clinical significance.

Thus far, DLN has been well studied in laryngeal cancer while its role in thyroid cancer remains unknown. The aim of this study was to assess the incidence and risk factors of DLN metastasis, and to develop a clinicopathologic characters-based diagnostic model to help surgeons predict preoperative DLN metastasis.

## Materials and Methods

### Patients

**Figure 1:**
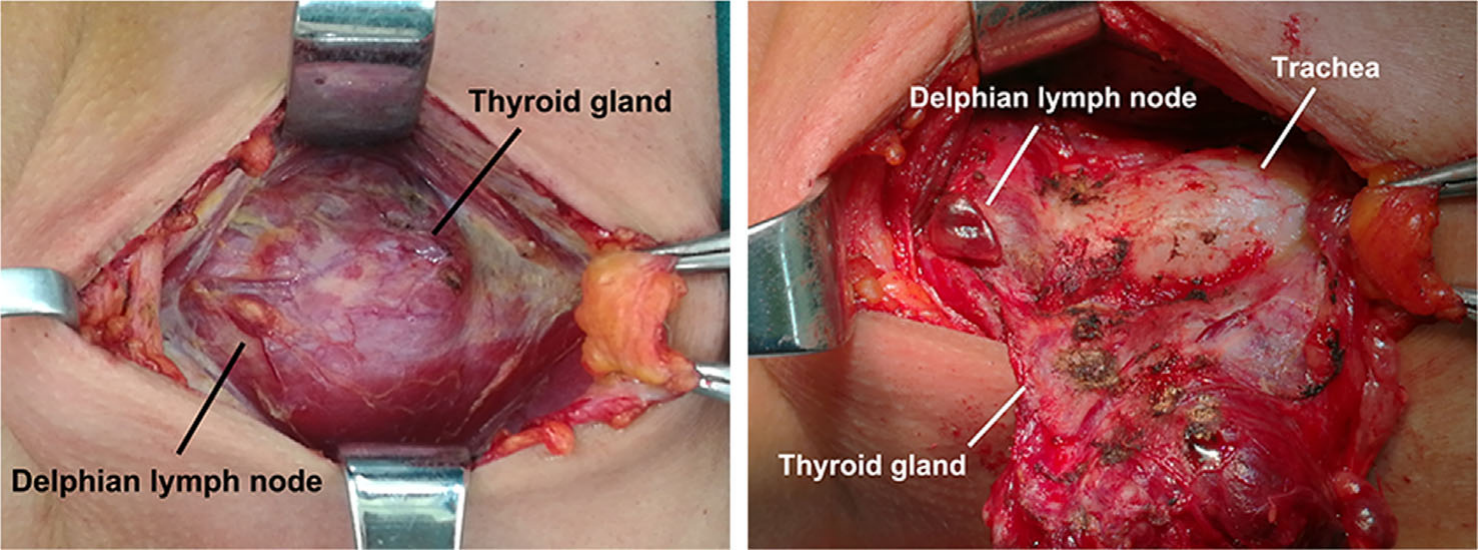
The Delphian lymph node (DLN).

We reviewed 936 patients who were diagnosed pathologically with PTC. All the patients underwent thyroidectomy by a single surgeon at the Head and Neck Surgery Department of Tianjin Cancer Hospital from June 2017 to January 2019. The precricoid region was deliberately excised and labeled as DLNs (**Figure 1**) and then diagnosed by histopathology. Overall, 250 patients who underwent thyroidectomy by another surgeon at Tianjin Cancer Hospital from January 2019 to April 2019 were used as a validation cohort to evaluate the performance of the diagnostic model. Of the 250 patients with PTC, 127 (50.8%) patients were found to have DLNs. DLN metastasis was observed in 28 (22.4%) patients. The mean number of metastatic DLNs was 1.89. The clinicopathologic information was gathered, and the protocol in this study was approved by the Tianjin Cancer Hospital Ethics Committee.

### Surgery

All the patients underwent thyroidectomy according to the Chinese practice guidelines in thyroid cancer (21, 22). For bilateral PTC patients, Total thyroidectomy was performed. For unilateral PTC patients, total thyroidectomy or lobectomy plus isthmusectomy were performed. When unilateral PTC patients met one of following conditions: tumor size >4 cm, multifocal in one lobe, extrathyroid invasion or distant metastasis, which were diagnosed by the use of frozen section analysis and preoperative examination, total thyroidectomy plus isthmusectomy may be considered, according to the guidelines of Chinese Thyroid Association. Meanwhile, pretracheal nodes, ipsolateral or bilateral paratracheal nodes, and peripheral fatty tissue were routinely dissected. The DLNs were removed once they were identified by the surgeon. Ipsilateral prophylactic central node dissection (pCND) was performed in cN0 PTC patients. When any of the cervical lymph nodes were verified as suspicious through preoperative ultrasonography, palpation or intraoperative inspection, a contralateral CND was performed. If lateral neck lymph node (Level II-V) was suspected with metastasis by preoperative ultrasound examination or affirmed with metastasis by fine needle aspiration, lateral node dissection (LND) would be performed.

### Statistical Analysis

The characteristics of patients were displayed as the mean ± SD or frequencies with percentages. The correlation between DLN metastasis and various clinical factors was analyzed by univariable analysis using Student’s *t*-test (for continuous variables) or the chi-squared test (for categorical variables). Logistic regression was used to identify the factors that were independently associated with DLN metastasis. The tolerance for all the potential predictors was > 0.1 and the variance inflation factor (VIF) was < 10. A multiple-variable logistic regression model, including all the variables with *P* values less than 0.05 in multivariate analysis, was used for translation into a nomogram diagnostic model. The model performance was assessed using discrimination and calibration (23). A nomogram was created to calculate individual probabilities (24). We evaluated the performance of the diagnosis model in the validation cohort and determined the means and 95% confidence intervals of the AUROC. The analyses were conducted using SAS version 9.4 (SAS Institute Inc., Cary, NC, USA) and R-software version 3.5.1 (R foundation for statistical computing, Vienna, Austria. URL http://www.R-project.org).

## Results

### Patient and Clinicopathologic Characteristics

Of all the 936 patients, DLNs were detected in 581 (62.07%, 581/936) patients, and the median numbers of DLNs were 2.1 (range, 1–10). In the group with identified DLNs, 177 (30.46%, 177/581) patients were confirmed to be DLN positive. The percentage of central lymph node metastasis in PTC patients was 59.40% (**Table 1**).

**Table 1:** Rates of Delphian lymph node (DLN) detection and metastasis.

Variables	N/total (%)*
DLN detection	581/936 (62.07%)
DLN metastasis	177/581 (30.46%)
Central neck node metastasis	556/936 (59.40%)
Mean no. of DLNs (range)	2.1 (1–10)
Mean no. of DLN metastases (range)	1.6 (1–9)

*Unless otherwise indicated.

The univariate analysis revealed that metastatic disease to the DLN was associated with male sex (52.5% vs. 22.2%, *p*<0.001), younger age at diagnosis (38.27 ± 11.997 vs. 43.86 ± 10.752, *p*<0.001), larger tumor (13.79 ± 8.8659 vs. 9.38 ± 4.3466, *p*<0.001), bilateral (39.0% vs. 24.9%, *p*<0.001), multifocality (46.3% vs. 30.7%, *p*<0.001), extrathyroid extension (ETE) (87.0% vs. 73.3%, *p*<0.001), lymphovascular invasion (10.7% vs. 1.5%, *p*<0.001), central neck node metastases (43.2% vs. 7.9%, *p*<0.001) and lateral neck node metastases (58.9% vs. 25.0%, *p*=0.022) (**Table 2**), whereas the correlations between DLN metastasis and thyroiditis (*p*=0.636) or tumor location (*p*=0.774) were not statistically significant.

**Table 2:** Comparison of clinicopathological characteristics between the patients with and without DLN metastasis.

Variable	No. of DLN-positive patients (%)	No. of DLN-negative patients (%)	*P* value
Gender			<0.001
Male	83 (46.89)	75 (18.56)	
Female	94 (53.11)	329 (81.44)	
Age (mean ± SD)	38.27 ± 11.997	43.86 ± 10.752	<0.001^*^
Thyroiditis			0.636
Yes	44 (24.85)	108 (26.73)	
No	133 (75.15)	296 (73.27)	
Tumor size (mean ± SD)	13.79 ± 8.8659	9.38 ± 4.3466	<0.001
Bilateral			
No	88 (49.71)	265 (65.59)	<0.001
Yes	89 (50.29)	139 (34.41)	
Multifocality	82 (46.32)	124 (30.69)	<0.001
Extrathyroid extension	154 (87.01)	296 (73.27)	<0.001
Lymphovascular invasion	19 (10.73)	6 (1.49)	<0.001
Location of tumor			
Solitary tumor	82 (46.33)	229 (56.68)	0.208^#^
Upper	16	47	
Middle-upper	11	42	
Middle	18	28	
Middle-lower	22	56	
Lower	15	56	
Multifocal tumor	95 (53.67)	175 (43.32)	0.109^&^
In one lobes	35	48	
In both lobes	60	127	
Central neck node metastases			<0.001
Present	161 (90.96)	212 (53.12)	
Absent	16 (9.04)	187 (46.88)	
Lateral neck node metastases			0.022
Present	103 (97.17)	72 (88.89)	
Absent	3 (2.83)	9 (11.11)	

Data are no. of patients (%) unless otherwise indicated.

*t test for tumor location analysis; ^#^for solitary tumors, upper vs. upper-middle vs. middle vs. middle-lower vs. lower; ^&^for multifocal tumor, both lobes vs. single lobe.

**Figure 2:**
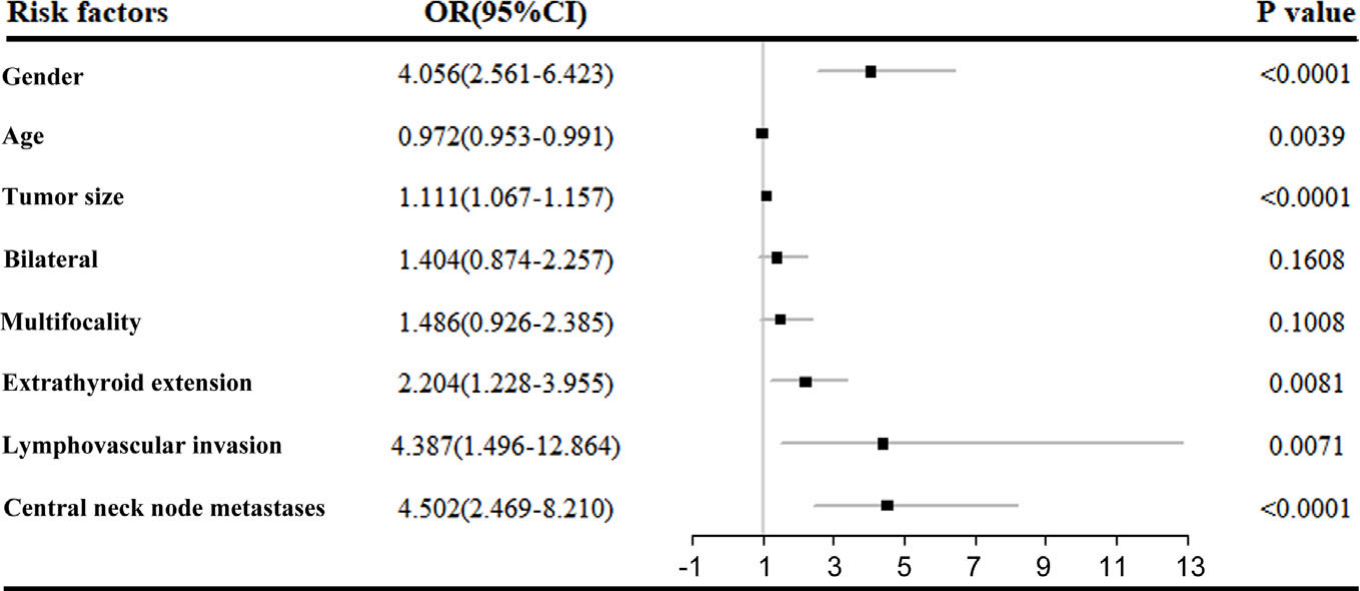
Multivariate logistic regression analysis of DLN metastasis.

All the variables with *P* values less than 0.05 in univariate analysis were selected for multivariate logistic analysis. As shown in **Figure 2**, gender (male, *p*<0.0001), age (younger age, *p*=0.0039), tumor size (>10mm, *p*<0.0001), ETE (*p*=0.0081), lymphovascular invasion (*p*=0.0071) and central neck node metastasis (*p*<0.0001) were independent risk factors of DLN metastasis. However, lateral neck lymph node metastasis was not included in the multivariate logistic analysis due to the limited number of cases. For central compartment metastasis, DLN status had a sensitivity, specificity, positive and negative predictive values of 43%, 92%, 91% and 47%, respectively. And for lateral lymph node metastasis, DLN metastasis had a respective sensitivity, specificity, positive and negative predictive values of 59%, 75%, 97% and 11% (**Table 3**).

**Table 3:** Ability of Delphian node metastasis to predict central and lateral lymph node metastasis.

Lymph node metastasis types	Sensitivity (%)	Specificity (%)	PPV (%)	NPV (%)	LR+	LR−
Central	43	92	91	47	5.38	0.62
Lateral	59	75	97	11	2.36	0.55

PPV, positive predictive value; NPV, negative predictive value;

LR+, positive likelihood radio; LR−, negative likelihood radio.

### Diagnostic Model Development and Its Validation

**Figure 3:**
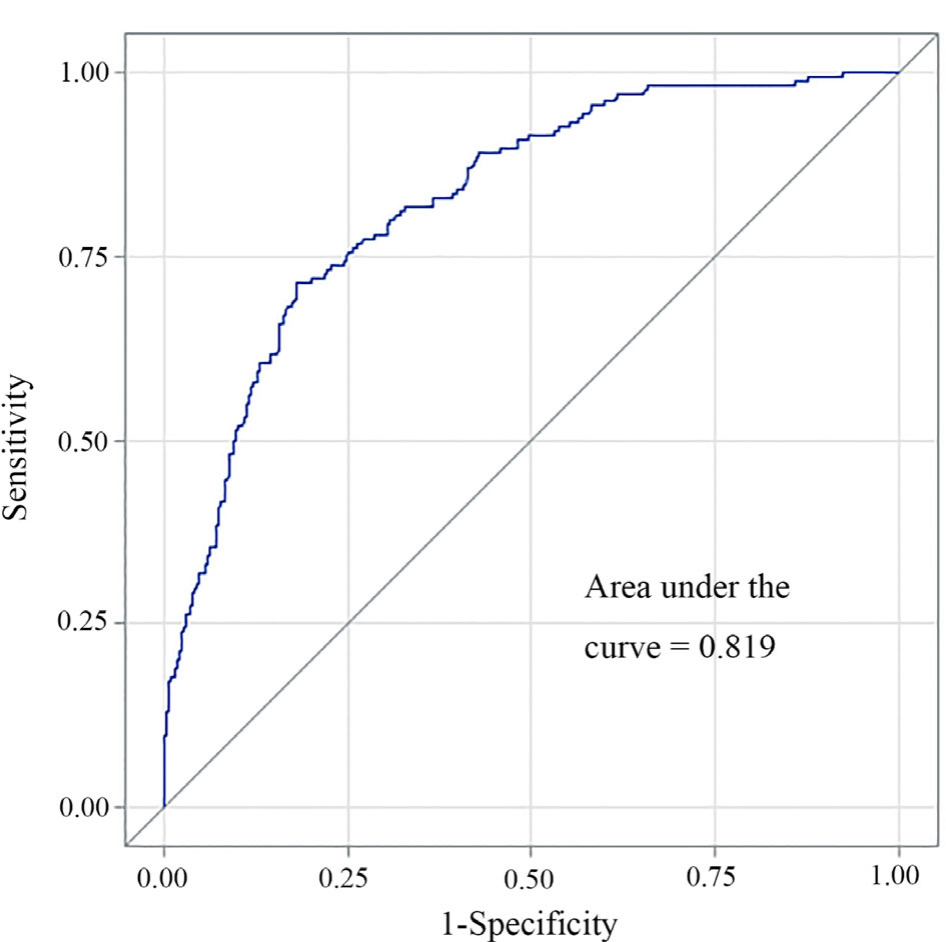
The AUC of the ROC curve of the proposed methods for the diagnosis of DLN metastasis.

**Figure 4:**
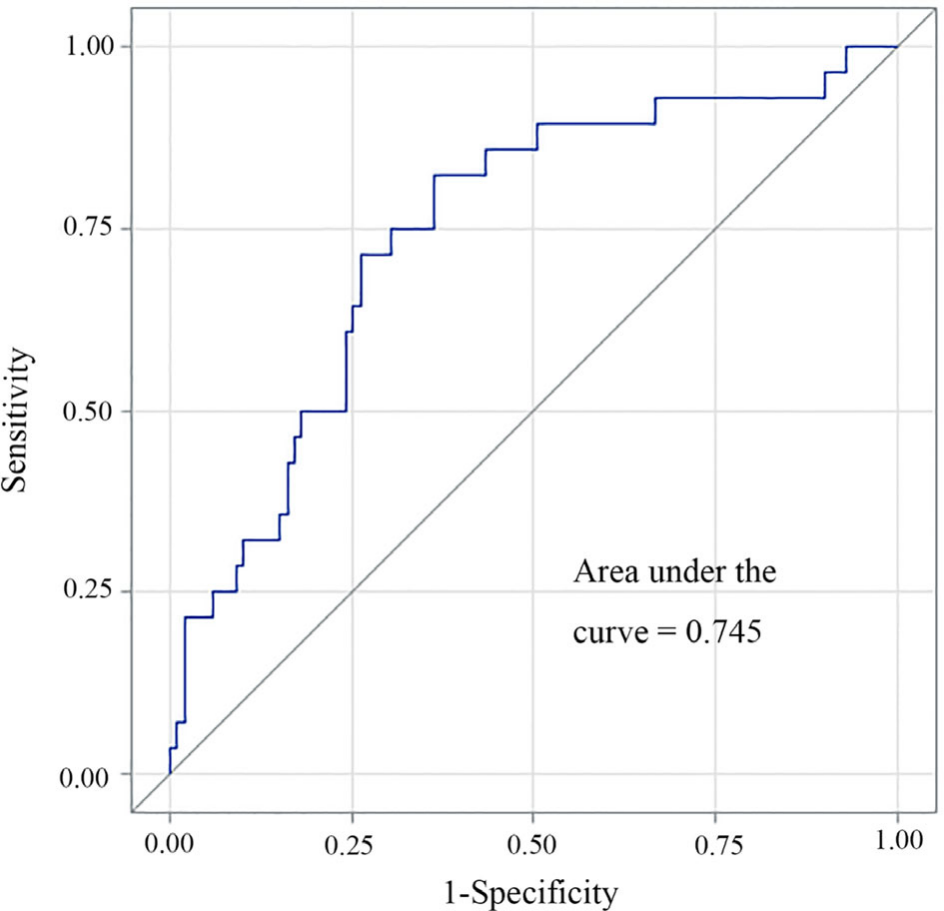
An external cohort verification of the prediction model for DLN metastasis.

**Figure 5:**
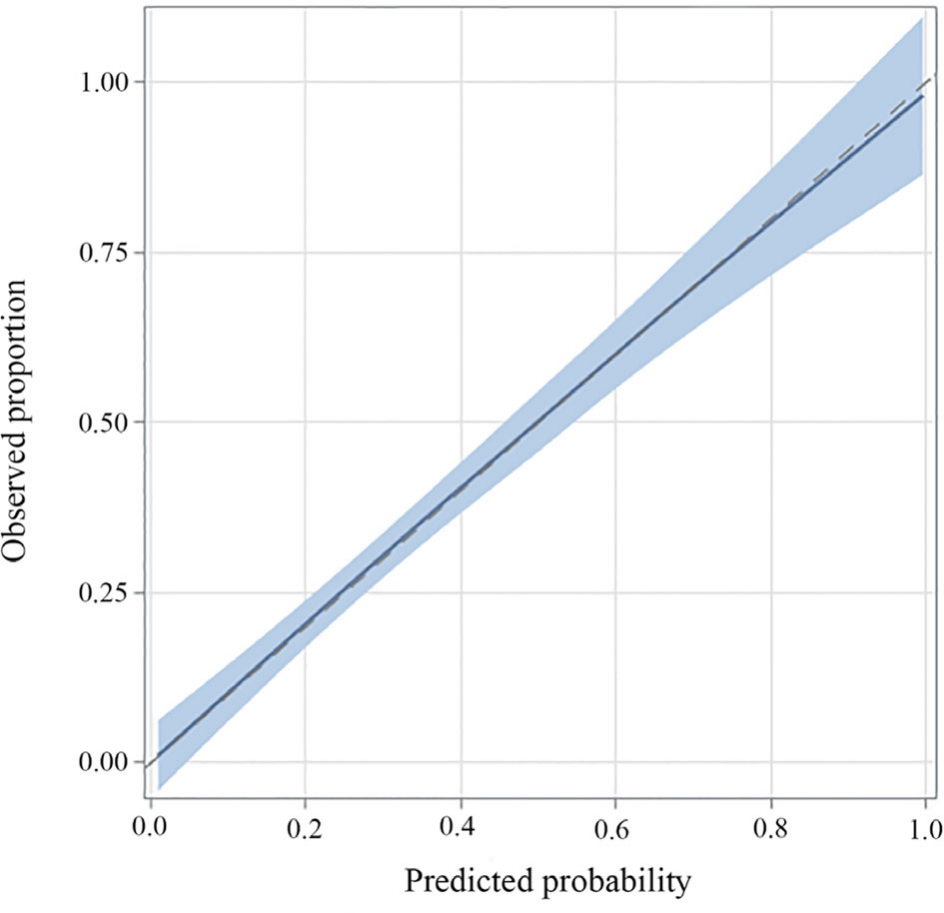
Calibration plots of recalibrated prognostic models to predict risk of DLN metastasis. In the case of perfect calibration, all the groups of predicted probabilities fit close to the blue diagonal line, corresponding to an intercept of 0 and a slope of 1 for the calibration plot. The vertical lines in grouped observations represent 95% confidence intervals.

A multivariate logistic regression model was adopted to establish the diagnostic model for DLN metastasis. Gender, age, tumor size, ETE, lymphovascular invasion and central neck node metastasis were included as the clinicopathologic features. For internal validation, the study population (n=556) was randomly assigned to training data sets (66%) or test data sets (33%). The diagnostic model was established with the training data while the test data were used to assess model performance with AUC. The area under the ROC curve (AUC) of the training data and testing data was 0.829 (95% CI, 0.804–0.854) and 0.819 (95% CI, 0.764–0.870), respectively (**Figures 3** and **4**). For external validation, 250 PTC patients who underwent thyroidectomy by another surgeon were used to evaluate the performance of the diagnostic model. The area under the ROC curve was 0.745. Calibration plots recalibrated the diagnostic models to predict risk of DLN metastasis. Calibration of the diagnosis model was satisfactory (**Figure 5**).

### Risk Factors-Based Nomogram Development

**Figure 6:**
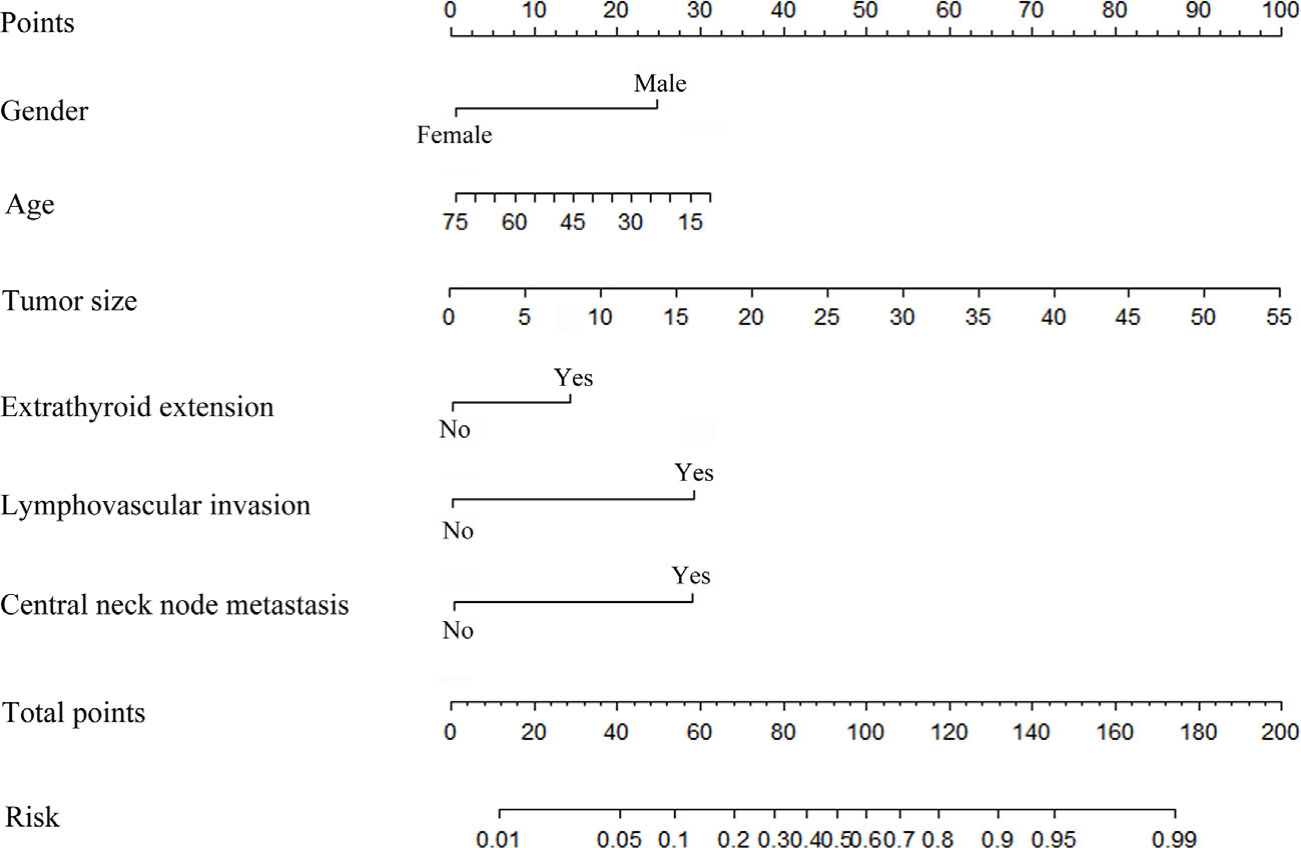
Risk factor-based nomogram for predicting DLN metastasis. Predictor points (“Points” scale; top) correspond to each variable.

A nomogram was developed to calculate the degree of individual risk in order to improve clinical diagnosis. The nomogram based on these significant variables was established (**Figure 6**). Among the six significant features, tumor size was one predominant predictor of DLN metastasis in the nomogram. When all these criteria were satisfied, the specificity was infinite close to 100%. For example, a 45-year-old man was diagnosed initially with PTC. His tumor size was 3 mm, and central lymph node metastasis on preoperative ultrasonography was positive. ETE was confirmed by intraoperative frozen biopsy, although there was no lymphovascular invasion. The DLNs were excised, and then, histopathological examination showed that the DLN had no metastasis. The probability of DLN metastasis assessed by nomogram was approximately 30%.

## Discussion

Delphian was first used as an eponym for the prelaryngeal node in thyroid disease by Raymond B. Randall, and it is hypothesized that metastasis to this lymph node predicts a poor prognosis from cancer (13, 20). Previous literature has reported that DLN positivity is a measurable parameter to predict extensive lymph node metastasis, recurrence and poor overall survival in laryngeal and hypopharyngeal cancer (25, 26). As for PTC, recent reports detected the DLN in 23% to 75% of PTC patients, and the DLN positivity rate was 8% to 25% (12, 13, 15, 16, 27–30). Metastasis to the DLN is associated with several clinicopathological characteristics of PTC patients, including age, gender, tumor size, tumor location, multifocality, ETE, lymphovascular invasion and central and lateral neck node metastasis (12–14, 16, 27, 28, 30). In our series, male PTC patients were more likely to have positive DLNs. In addition, the rate of positive DLNs in younger patients was higher than that in elderly patients, suggesting that age was negatively correlated with DLN metastasis. Crucially, several adverse prognostic factors in PTC, including ETE, increased volume (number and size) of primary tumor, lymphovascular invasion and central and lateral node metastasis were verified to be positively related to DLN metastasis. Multivariable analyses revealed that gender, age, tumor size, ETE, lymphovascular invasion and central neck node metastasis were independent risk factors.

The DLN receives afferent lymph flow from the larynx and thyroid gland, which then flows towards the central and lateral neck lymph nodes (12). Studies have reported that patients with DLN metastasis are five to eight times more likely to have central compartment disease and 3.5 to 4 times more likely to have lateral neck lymph node metastasis (12–14). DLN positivity is predictive of further central and lateral lymph node metastasis (20). Hence, once metastatic disease to the DLN is identified, the surgeon should pay greater attention to the central and lateral neck compartment. We confirmed that the metastatic rate to the central lymph node and lateral neck lymph node in DLN-positive patients was 161 of 177 (90.96%) and 103/106 (97.17%), respectively. There has been debate over many years regarding whether prophylactic central node dissection should be performed in clinically N0 (cN0) PTC patients, and the prognostic significance of metastasis in this node group is controversial. Although NCCN clinical practice guideline in thyroid carcinoma and the American Thyroid Association guidelines no longer recommend prophylactic central compartment clearance in all cN0 PTC patients (31, 32), the Chinese Thyroid Association guidelines (21, 22) still recommend prophylactic central neck dissection, because the occult neck lymph node metastasis rate in cN0 PTC is still up to 72% (14), which could be the seeds of recurrence. Therefore, the central compartment should still be critically evaluated in all patients with cN0 PTC. The DLN, as one component of Level VI, is relatively sensitive for estimating Level VI metastatic disease. Joseph D. et al. (13) investigated 103 patients with thyroid cancer, and 21.4% of the patients were DLN positive. In that analysis, DLN involvement was associated with greater nodal disease (9.8 vs. 1.6 nodes), larger tumor size (19.4 mm vs. 11.1 mm) and younger age (41 vs. 47 years). Importantly, these anthors confirmed that DLN positivity remarkably predicted further disease in the neck lymph nodes. DLN-positive patients were approximately 8 times, 4 times and 60 times more likely to have central node disease, lateral node disease, and any neck nodal disease respectively. Our results were similar to the data mentioned above. Additionally, in our study, the diameter of the tumor was more than 5mm in 96.05% (170/177) of DLN-positive PTC and more than 10 mm in 66.67% (118/177) of DLN-positive PTC patients. Moreover, our results showed that DLN involvement was predictive of further disease in the central lymph node (sensitivity: 41%, specificity: 92%, positive predictive value (PPV): 91% and negative predictive value (NPV): 60%) and moderately predictive of further disease in the lateral neck compartment (sensitivity: 59%, specificity: 75%, PPV: 97%, NPV: 11%). Collectively, evaluating the status of the DLN is beneficial for the selection of lymph node management. Based on these data, once the status of DLN is sensitively evaluated, we can better predict the criticality of lymph node involvement.

In our study, conclusive evidence of DLN metastasis was obtained by a nomogram that was developed according to all adverse factors. These factors greatly contribute to a high risk of disease metastasis and recurrence (31). Ideally, a more accurate measurement before the operation and evaluation of the frozen sections of samples collected during the operation is essential for diagnosing DLN and Level VI nodal metastases. The nomogram proposed in this study, incorporating independent risk factors, provides a useful tool for surgeons to improve metastatic disease prediction and decision-making. To the best of our knowledge, this is the first retrospective study in PTC patients to search for clinicopathologic risk factors and further develop a diagnostic model for DLN metastasis. However, further studies should be conducted to validate the potential application value of nomograms in PTC patients with suspicious or confirmed DLN metastasis. Admittedly, our study was inherently limited by the retrospective single-center design with a probable selection bias. Moreover, lateral lymph node dissection was performed only when there was evidence of metastasis, which resulted in insufficient enrollment of negative cases. Hence, we excluded lateral lymph node metastasis from the multivariable analyses and the significance of lateral neck node metastasis might be underestimated.

In conclusion, our findings demonstrate that metastatic PTC to the DLN is associated with a number of clinicopathologic characteristics. As the DLN could be a clinical sensitive predictor of further neck lymph nodes, particularly the central compartment, we developed a diagnostic model that includs all adverse factors. If there is strong suspicion of metastatic DLN disease in cN0 PTC, the remainder of the central compartment should be evaluated for further nodal metastasis, and the appropriate dissection should be performed.

## Data Availability Statement

The raw data supporting the conclusions of this article will be made available by the authors, without undue reservation.

## Ethics Statement

This study was approved by the Medical Ethics Committee of the Tianjin Medical University Cancer Institute & Hospital (bc2020046), and written informed consent was obtained from each patient.

## Author Contributions

CJ and YSD conceived and designed the study. XCL, DDL, and HWL collected the clinical information and performed most of the statistical analyses. MQZ, KY, YJS, and YW contributed to the data analysis and interpretation. XDW and YSW provided clinical samples and information. The manuscript was written by XCL and revised by CJ and YSD. CYJ contributed to the revision of the manuscript. CJ, XDW, and YSW supervised the research. All authors contributed to the article and approved the submitted version.

## Conflict of Interest

The authors declare that the research was conducted in the absence of any commercial or financial relationships that could be construed as a potential conflict of interest.
